# Circ_0004087 interaction with SND1 promotes docetaxel resistance in prostate cancer by boosting the mitosis error correction mechanism

**DOI:** 10.1186/s13046-022-02404-3

**Published:** 2022-06-03

**Authors:** Liang Chen, Yarong Song, Teng Hou, Xuexiang Li, Lulin Cheng, Yunxue Li, Yifei Xing

**Affiliations:** Department of Urology, Union Hospital, Tongji Medical College, Huazhong University of Science and Technology, Wuhan, 430022 Hubei Province China

**Keywords:** Circ_0004087, Docetaxel resistance, Mitosis error correction mechanism, Chromosomal passenger complex, Prostate cancer

## Abstract

**Background:**

Acquisition of the chemoresistance to docetaxel (DTX), a microtubule-targeting agent, has been a huge obstacle in treatment for metastatic castration-resistant prostate cancer (mCRPC). Recently, strategies targeting the mitosis error correction mechanism including chromosomal passenger complex (CPC) were reported to reverse the resistance to microtubule-targeting anticancer agents. Meanwhile, accumulating evidence indicated the important roles of circRNAs in DTX resistance of prostate cancer (PCa). However, whether circRNAs could regulate DTX chemosensitivity by affecting the mitosis error correction mechanism remains unclear.

**Methods:**

Expression patterns of circ_0004087 and BUB1 were determined through mining the public circRNA datasets and performing western blot and qRT-PCR assays. Agarose gel electrophoresis, Sanger sequencing, and RNase R treatment were conducted to examine the circular characteristics of circ_0004087. CircRNA pull-down, mass spectrometry analysis, Co-IP, and dual-luciferase reporter assays were performed to uncover the interaction among circ_0004087, SND1, and MYB. The effects of circ_0004087 and BUB1 on docetaxel-based chemotherapy were explored by flow cytometry and in vivo drug studies upon xenografted tumor model.

**Results:**

In the present study, we revealed the profound interaction between a novel circRNA, circ_0004087, and the mitosis error correction mechanism. Mechanistically, circ_0004087 binding with transcriptional coactivator SND1 could stimulate the transactivation of MYB and enhance the expression of downstream target BUB1. In turn, elevated BUB1 expression further recruited CPC to centromeres and guaranteed the error-free mitosis of PCa cells. Biologically, the overexpression of circ_0004087 conferred while the knockdown impaired DTX resistance in PCa cells.

**Conclusions:**

Our study uncovered the crucial role of circ_0004087/SND1/MYB/BUB1 axis in modulating the error mitosis correction mechanism and DTX chemoresistance, suggesting that circ_0004087 may serve as a valuable prognostic biomarker and a potential therapeutic target in DTX-resistant PCa patients.

**Supplementary Information:**

The online version contains supplementary material available at 10.1186/s13046-022-02404-3.

## Background

Prostate cancer has been reported as the second most commonly diagnosed malignancy in men and the fifth leading cause of cancer-related death worldwide [1]. Worse still, despite the initial sensitivity to androgen deprivation therapy, most cases inevitably progress into CRPC, a fatal stage of PCa [2]. As one of the taxol-derived drugs, DTX is regarded as the standard first-line chemotherapy in mCRPC patients [3]. Nevertheless, the lengthening of survival time is moderate due to the inevitable acquisition of the drug resistance [4–6]. Thus, it is of great priority to clarify the underlying molecular mechanisms involving in DTX resistance and identify the promising therapeutic targets.

CircRNAs are a kind of single-stranded RNAs and covalently bonded at the 3’ and 5’ ends in a closed loop [7]. It has been wildly reported that circRNAs are highly conserved, resistant to the digestion of exonuclease RNase R and characterized by the tissue-specific expression patterns [8, 9]. Some of them function as miRNA sponges to modulate genes expression in a post-transcriptional way [10, 11]. Besides, some circRNAs cooperate with partner proteins to form circRNA-protein complexes (circRNPs) and subsequently regulate functions of associated proteins [12–14]. Some aberrantly expressed circRNAs were found to be involved in the development of chemoresistance in prostate cancer as miRNA sponge [15–17], however, little is known about whether circRNAs could form circRNPs to regulate the DTX resistance in prostate cancer.

It is well established that the chromosomal passenger complex (CPC), consisting of INCENP, survivin, borealin, and Aurora kinase B (AURKB), contributes to the correction of misaligned chromosomes during mitosis and play a crucial role in error-free chromosome segregation [18–20]. Lately, a novel approach to cancer treatment was put forward: suppressing the repair mechanism of cell-cycle can bring lethal-degree damage to tumor cells, thus becoming a promising way of tumor therapy [21, 22]. Furthermore, strategies targeting the mitosis correction mechanism, such as CPC, were proved to augment the effect of microtubule-targeting anticancer agents and reverse the drug resistance [22, 23]. These researches suggested that mitosis error correction mechanisms like CPC might be effective therapy targets of tumors in drug-resistant stage.

In the present study, we identified circ_0004087, derived from exon 2 of the CDYL2 gene, being upregulated in prostate cancer. Following studies revealed that circ_0004087 could interact with SND1 to facilitate DTX chemoresistance of prostate cancer by promoting the mitosis error correction function of CPC. Moreover, downregulating circ_0004087 in DTX-resistant PC-3 cells (PC-3-DR) could partly attenuate its drug resistance ability. Our findings disclosed the essential role of circ_0004087 in DTX resistance and provided a promising target for treatment of prostate cancer in chemoresistance stage.

## Methods

### Clinical tissue specimens

Specimens of PCa tissues and adjacent noncancerous tissues were obtained from patients who underwent radical prostatectomy at Union Hospital of Tongji Medical College of Huazhong University of Science and Technology (Wuhan, China) from 2015 to 2019. All cases were confirmed by clinical and pathological diagnosis. All patients provided written informed consent. Human tissue study was carried out in accordance with the Declaration of Helsinki and approved by the Ethics Committee of Union Hospital of Huazhong University of Science and Technology.

### Cell culture

Human prostatic epithelial cell line (RWPE-1) and PCa cell lines 22Rv1, PC-3, and DU145 were purchased from the Cell Bank of the Chinese Academy of Sciences (Shanghai, China). PCa cell lines LNCaP and C4-2 were gifted from Prof. Jun Zhao and Prof. Xiaoping Zhang (Union Hospital, Wuhan, China). All PCa cells were cultured in RPMI-1640 medium, supplemented with 10% fetal bovine serum (FBS). RWPE-1 cell line was cultured in Prostate Epithelial Cell Medium (PEpiCM) (ScienCell, CA, USA). All cells used in this study were maintained in a 5% CO_2_ incubator at 37 °C.

### Quantitative real-time PCR (qRT-PCR)

Total RNA that extracted from cell lines or fresh tissues with TRIzol reagent (Invitrogen, CA, USA) was reverse transcribed into cDNA using PrimeScript RT Reagent Kit (Takara, Japan). To quantify the expression level, qRT-PCR was performed on a StepOnePlus Real-Time PCR System (Applied Biosystems, CA, USA). 2^–ΔΔCT^ method was used to calculated the relative levels of circRNA and mRNA. GAPDH and U6 were used as internal controls. Primers used in this study were synthesized by Sangon Biotech (Shanghai, China) (Additional file 1).

### Western blot analysis

Proteins of cells or tissues were extracted with RIPA buffer and the concentration was measured using BCA Protein Assay Kit (Thermo Scientific, MA, USA). Protein samples were separated in 8–12% SDS-PAGE gels and then transferred onto PVDF membranes. Afterward, membranes were blocked for 1 h and incubated with primary antibodies overnight. After the incubation with the corresponding species-specific secondary antibodies for 1 h, bands were detected by chemiluminescence using an electrochemiluminescence (ECL) system. Primary antibodies used in this study are as follows: anti-Alpha Tubulin (66,031–1-Ig, Proteintech, USA), anti-SND1 (60,265–1-Ig, Proteintech, USA), anti-C-Myb (17,800–1-AP, Proteintech, USA), anti-BUB1 (13,330–1-AP, Proteintech, USA), anti-Histone H2A (ab18255, Abcam, UK), anti-p-Histone H2A (ab177391, Abcam, UK).

### Plasmids construction and cell transfection

Full length circ_0004087 was synthesized, and inserted into GV486 vector to construct the overexpression plasmid (GeneChem, Shanghai, China). Small hairpin RNAs (shRNAs) specifically targeting circ_0004087 were designed, and cloned into GV248 vector to construct knockdown plasmids (GeneChem, Shanghai, China). Lipofectamine 2000 Transfection Reagent (Invitrogen, CA, USA) was used to perform the plasmids transfection. Stable cell lines were selected with G418 or puromycin for at least 30 days.

### RNA fluorescence in situ hybridization (RNA-FISH)

RNA-FISH assay was performed upon PCa cell lines with Fluorescent In Situ Hybridization Kit (RiboBio, Guangzhou, China). Specific RNA-FISH probes for circ_0004087, 18S, and U6 were synthesized by RiboBio (Guangzhou, China). Briefly, cells were fixed by 4% paraformaldehyde and subsequently permeabilized with 0.5% Triton X-100. After being hybridized with the specific probes, cells were counterstained with DAPI. Finally, all fluorescence images were captured with a Nikon A1R-si Laser Scanning Confocal Microscope (Nikon, Japan).

### Pull-down assay with biotinylated circRNA probe

For pull-down assay, 10^7^ cells were collected and lysed in lysis buffer (50 mM Tris–HCl pH 7.5, 150 mM KCl, 1 mM EDTA, and 0.5% Triton X-100 supplemented with 1 × proteinase inhibitor cocktail). Lysate was hybridized with the biotin-labeled circ_0004087 probe and the corresponding control probe for 4–6 h. All samples were then incubated with the M-280 streptavidin magnetic beads (Invitrogen, CA, USA) at 4℃ overnight. The following day, beads were washed and resuspended. Lastly, samples were heated with 1 × Loading Buffer at 95 °C for 10 min. The sequences of probes are listed in Additional file 1.

### Xenografted tumor model

Male BALB/c nude mice (3–5 weeks) were purchased from the Center of Experimental Animals of Tongji Medical College. To construct the subcutaneous xenografted tumor model, approximately 2 × 10^6^ PCa cells were injected subcutaneously into the dorsal flanks of mice. Based on the experimental requirement, 10 mg/kg DTX or DMSO in PBS were administered intraperitoneally once per week. Tumor volumes were calculated using the equation (L × W^2^)/2 every third day. On day 30, mice were anesthetized and euthanized, tumors were excised and weighed. Images of xenografts were captured using the In Vivo Optical Imaging System (In Vivo FX PRO, Bruker, MA, USA) or the digital single-lens reflex camera (D610, Nikon, Japan). All experiments related to animals in this study were approved by the Ethics Committee of Union Hospital of Huazhong University of Science and Technology.

### Apoptosis assay

To measure the apoptotic rate, PCa cells were seeded into six-well plates. Annexin V-PE/7-AAD Apoptosis Detection Kit (Vazyme, Nanjing, China) was used based on the manufacturer’s instruction. In brief, cells were washed twice in ice-cold PBS, and incubated with 100 μl 1 × Binding Buffer supplemented with 5 μl Annexin V-PE and 5 μl 7-AAD Staining Solution. 10 min later, another 400 μl 1 × Binding Buffer were added. Apoptotic rate was measured by flow cytometry, and data was analyzed by FlowJo software.

### Co-immunoprecipitation (Co-IP) assay

For Co-IP assay, approximately 10^7^ cells were collected and lysed in IP buffer (50 mM Tris–HCl PH 7.4, 150 mM NaCl, 2 mM EDTA, and 1% NP-40 supplemented with 1 × proteinase inhibitor cocktail and 1 mM PMSF) for 30 min on ice. Then, primary antibodies were added into the supernatant of the lysate acquired above. After incubating for 2 h, Protein A/G Magnetic Beads were added to samples in each group. Finally, uncombined matters were removed, beads were resuspended with 2 × Loading Buffer and boiled at 95 °C for 10 min. The following antibodies were used for Co-IP assay: anti-SND1 (60,265–1-Ig, Proteintech, USA), anti-C-Myb (17,800–1-AP, Proteintech, USA), anti-Mouse IgG (ab37355, Abcam, UK), anti-Rabbit IgG (ab171870, Abcam, UK).

### Statistical analysis

Statistical analysis in this study was conducted with SPSS and Prism software. Survival curves were calculated by the Kaplan–Meier method and compared using the log-rank test. Differences between groups were analyzed using the two-tailed Student’s t test. In this study, data are presented as the mean ± SD of three independent experiments, and *P* < 0.05 was considered statistically significant.

## Results

### Circ_0004087 is aberrantly overexpressed in prostate cancer

**Fig. 1:**
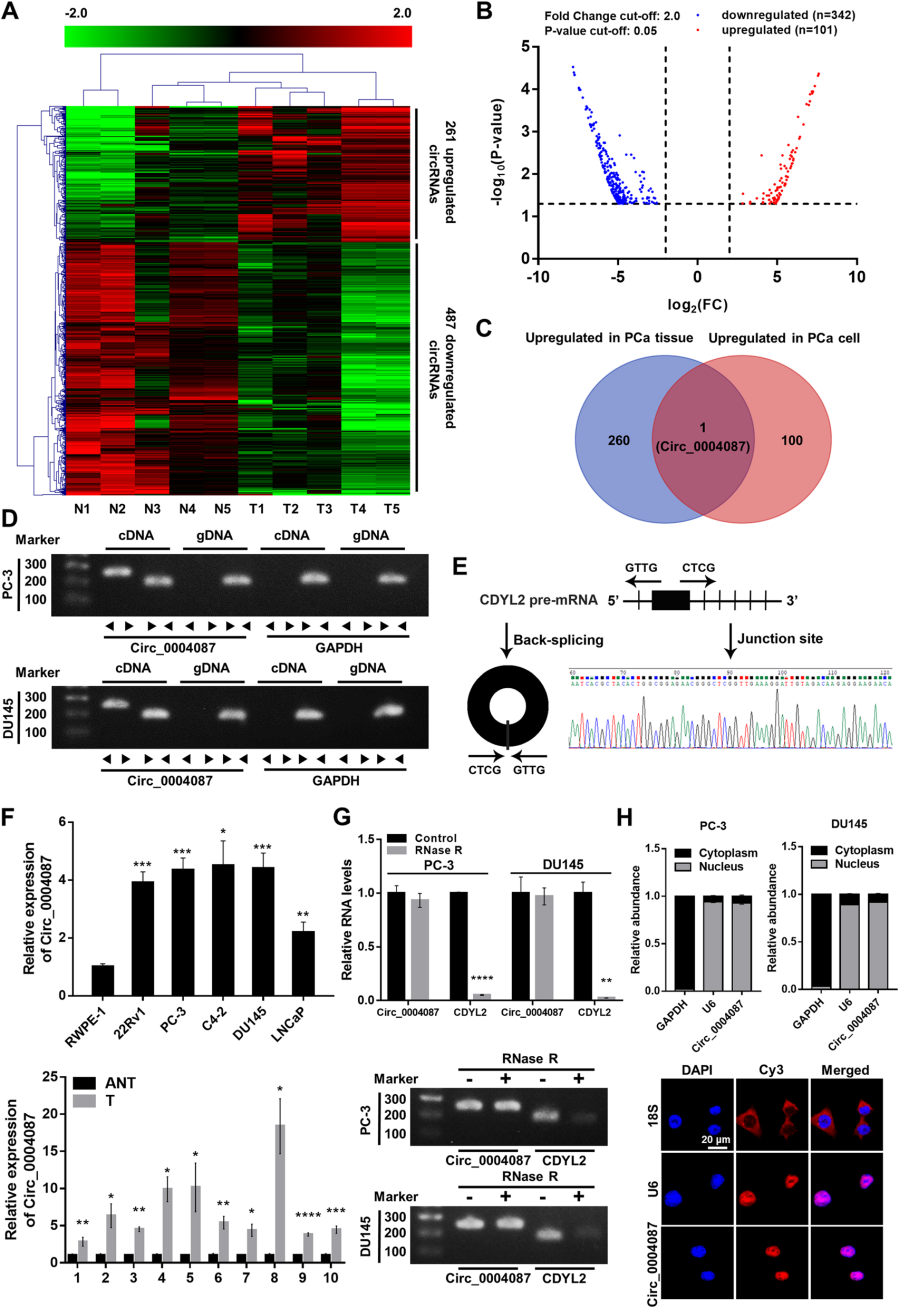
Circ_0004087 is significantly upregulated in PCa. **(A)** The heatmap of circRNA expression pattern in PCa tissue generated from microarray profile. **(B)** The differentially expressed circRNAs in PCa cell line identified by RNA-seq data. |log_2_(FC)|≥ 2, *P* < 0.05. **(C)** The venn diagram showing the intersection of the microarray profile and the RNA-seq data. **(D)** The existence of circ_0004087 in cDNA or genomic DNA (gDNA) of PCa cell lines determined using divergent and convergent primers. GAPDH was used as a negative control. **(E)** Schematic illustration showing the genomic location and the junction site of circ_0004087. **(F)** (Top) Relative levels of circ_0004087 in human prostatic epithelial cell line (RWPE-1) and PCa cell lines (22Rv1, PC-3, C4-2, DU145, and LNCaP). (Bottom) Relative levels of circ_0004087 in 10 pairs of PCa and adjacent normal tissues. ANT, Adjacent normal tissues; T, Tumor tissues. **(G)** The expression of circ_0004087 and its linear counterpart CDYL2 after RNase R treatment in PC-3 and DU145 cells. **(H)** RNA nucleus/cytoplasm separation assay (top) and RNA-FISH assay (bottom) revealing the distribution of circ_0004087 in PCa cell. 18S and GAPDH were applied as positive controls in cytoplasm, U6 was applied as a positive control in nucleus. Circ_0004087, 18S, and U6 probes were labeled with Cy3, nuclei were stained with DAPI. 1600 × . Statistical analysis is shown on the bar graphs. Data are presented as the mean ± SD of three independent experiments. * *P* < 0.05, ** *P* < 0.01, *** *P* < 0.001, **** *P* < 0.0001

To explore the expression pattern of circRNAs in PCa, we mined the microarray profile [24] and the RNA-seq data [25] from two published researches, and identified 261 and 101 upregulated [log_2_(FC) ≥ 2, *P* < 0.05] circRNAs respectively, in PCa tissue (Fig. 1A) and cell line (Fig. 1B). Among them, only circ_0004087 was upregulated in both cancerous tissue and cell (Fig. 1C). Then, the existence of this 592-nt circRNA generated from exon 2 of CDYL2 pre-mRNA was confirmed by PCR with divergent primers (Fig. 1D) and Sanger sequencing (Fig. 1E). To further verify the aberrant upregulation of circ_0004087 in PCa, qRT-PCR assay was performed. As expected, results were consistent with the expression matrix generated from microarray profile and the RNA-seq data (Fig. 1F). Moreover, the fact that circ_0004087 showed stronger resistance to RNase R than its linear counterpart reflected its circular characteristic of exonuclease degradation resistance (Fig. 1G).

For the reason that it is broadly accepted that the function of circRNA is closely related to the subcellular localization [26], we experimented with the RNA nucleus/cytoplasm separation and RNA-FISH assays. The results revealed the intra-nucleus distribution of circ_0004087 (Fig. 1H).

### Circ_0004087 positively modulates the resistance of prostate cancer cells to docetaxel

**Fig. 2:**
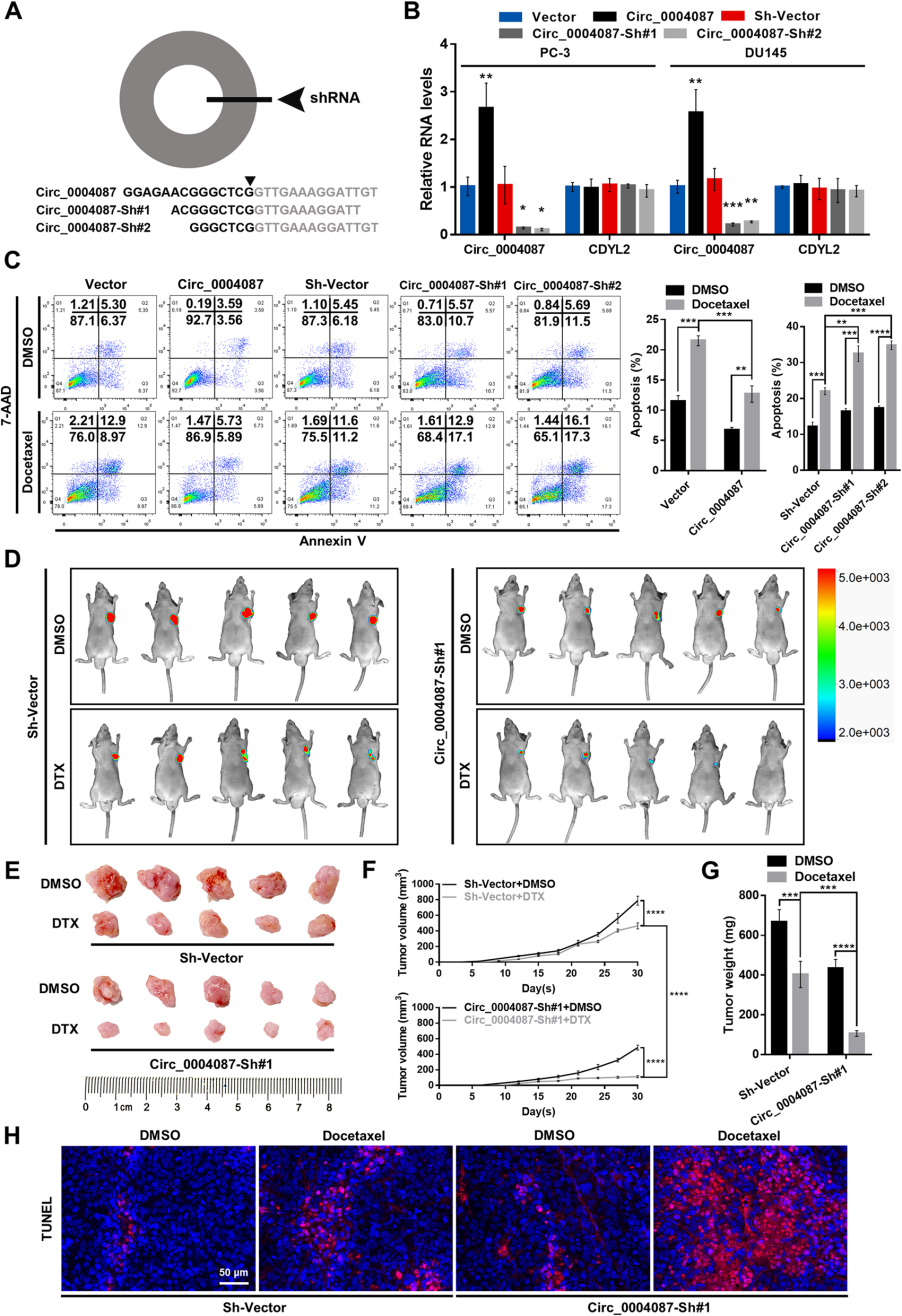
Circ_0004087 promotes the docetaxel resistance of PCa cells. **(A)** Schematic representation of the sites targeted by shRNAs designed for circ_0004087. **(B)** The levels of circ_0004087 and CDYL2 in the indicated stable cell lines. **(C)** The apoptosis rate of PCa cells expressing different circ_0004087 levels measured by flow cytometry. Cells were treated with 5 nM DTX for 48 h in this experiment. Cells went through the early apoptosis were marked with Annexin V-PE^+^/7-AAD^−^, cells went through the late apoptosis were marked with Annexin V-PE^+^/7-AAD^+^. **(D)** Fluorescent images of xenografts in nude mice acquired by an in vivo optical imaging system. **(E)** Images of excised xenografts acquired using the digital single-lens reflex camera. **(F)** Tumor volumes were measured every third day. **(G)** Tumor weights were measured on the day 30. **(H)** Immunohistochemical images of TUNEL staining on xenografts from each group 400 × . Statistical analysis is shown on the bar graphs. Data are presented as the mean ± SD of three independent experiments. * P < 0.05, ** *P* < 0.01, *** *P* < 0.001, **** *P* < 0.0001

Given that the extensive usage of DTX in clinical practice, we then examined the potential effect of circ_0004087 on DTX sensitivity in PCa cells. Circ_0004087 knockdown was accomplished with independent small hairpin RNAs (shRNAs) designed against the junction site (Fig. 2A). After the selection with specific antibiotics, the levels of circ_0004087 and CDYL2 in overexpressing cell lines (Circ_0004087) and knockdown cell lines (Circ_0004087-Sh#1 and Circ_0004087-Sh#2) were determined (Fig. 2B). As measured by flow cytometry, overexpression of circ_0004087 enhanced, while knockdown of circ_0004087 attenuated DTX resistance in PCa cells (Fig. 2C).

To explore the impact of circ_0004087 on DTX sensitivity in vivo, the subcutaneous xenograft tumor model was established. Consistent with the results in vitro, athymic nude mice subcutaneously injected with the circ_0004087-knockdown cells exhibited a significant decrease in DTX resistance (Fig. 2D, E, F and G). Expectedly, a more intense TUNEL staining was observed in circ_0004087-knockdown group after the administration of DTX (Fig. 2H).

Together, these results suggested that circ_0004087 promotes the DTX resistance of PCa cells in vivo and in vitro.

### Circ_0004087 interacts with SND1 protein in prostate cancer cells

**Fig. 3:**
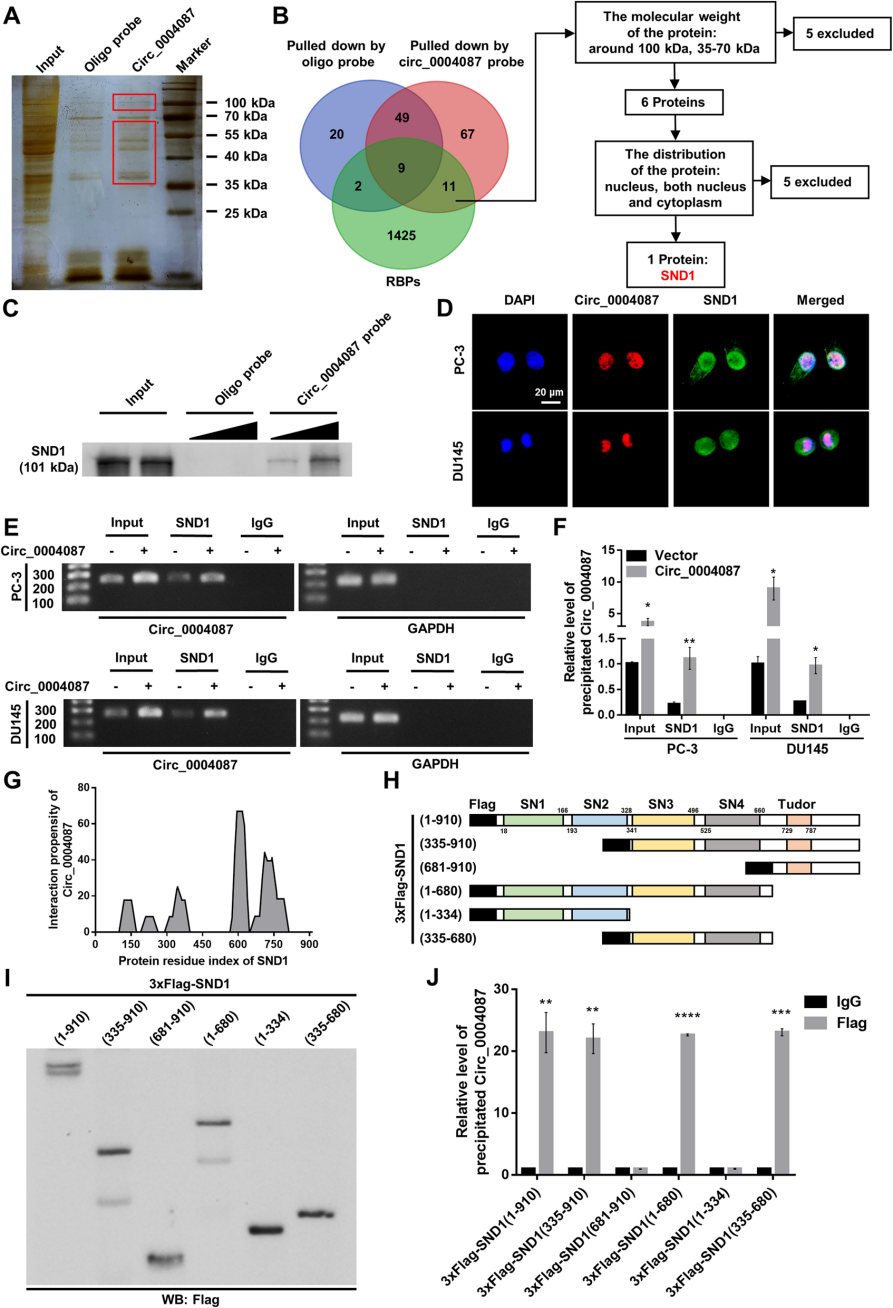
Circ_0004087 interacts with SND1 in PCa cells. (**A)** Silver staining showing proteins that interact with circ_0004087 in PCa cells. Red boxes show the location of differential bands. **(B)** The flow chart for screening out the protein partner of circ_0004087. **(C)** Western blot verifying the protein pulled down by biotin-labeled circ_0004087 probe. **(D)** RNA-FISH and immunofluorescence staining assays exhibiting the subcellular localization of circ_0004087 (red) and SND1 (green) in PCa cell lines. Nuclei were stained with DAPI 1600 × . **(E)** The result of RIP assay with anti-SND1 antibody showed by agarose gel electrophoresis diagram. IgG was used as a negative control. **(F)** Quantification of the result of RIP assay with anti-SND1 antibody. IgG was used as a negative control. **(G)** The binding propensity between circ_0004087 and SND1 protein predicted by the catRAPID algorithm. **(H)** Structure diagrams showing full-length and truncated Flag-tagged recombinant SND1 proteins. **(I)** Western blot validating the full-length and truncated Flag-tagged recombinant SND1 proteins. **(J)** RIP assay with anti-Flag antibody revealing the binding domains of SND1 to circ_0004087. IgG was used as a negative control. Statistical analysis is shown on the bar graphs. Data are presented as the mean ± SD of three independent experiments. * *P* < 0.05, ** *P* < 0.01, *** *P* < 0.001, **** *P* < 0.0001

Recently, many circRNAs located in the nucleus were proved to interact with different proteins, and subsequently influence the functions of the associated proteins [12–14, 27]. To identify the protein partners of circ_0004087, we synthesized the biotin-labeled probe specifically targeting the junction site of circ_0004087 (Additional file 2A). The efficiency and specificity of this biotin-labeled probe were confirmed firstly (Additional file 2B and Additional file 2C), then the pull-down assay was performed. The sensitive silver staining for precipitated samples demonstrates that the molecular weight of potential protein partners was about 100 kDa or between 35 and 70 kDa (Fig. 3A). Overlapping the result of mass spectrometry assay (GeneCreate, Wuhan, China) (Additional file 3) with the established RNA-binding proteins (RBPs, https://www.ablife.cc) (Additional file 4) identified 11 RBPs which were specifically pulled down by circ_0004087. Among them, only SND1 met the criteria: a. proteins with a weight around 100 kDa or between 35 and 70 kDa, b. proteins located in nucleus or both nucleus and cytoplasm of PCa cells (Fig. 3B and Additional file 2D). Validating biotin-labeled RNA pull-down assay further indicated that circ_0004087 was dose-dependently interactive with SND1 (Fig. 3C). Besides, RNA-FISH and immunofluorescence (IF) assays confirmed the co-localization of circ_0004087 and SND1 in PCa cells (Fig. 3D). Consistent with the RNA pull-down assay, RIP assay with SND1 antibody uncovered the combination between circ_0004087 and SND1 as well (Fig. 3E and F).

To precisely depict the structural determinants of the interactions between circ_0004087 and SND1, we first predicted the binding residue of SND1 protein using the catRAPID algorithm [28] (Fig. 3G and Additional file 5) and then verified the prediction by constructing multiple Flag-tagged protein truncations of SND1 (Fig. 3H and I). The results of in silico algorithm and in vitro binding assay both suggested the highest interaction propensity of SN3 and SN4 domains to circ_0004087 (Fig. 3J), which was consistent with the previous study [29]. Notably, the levels of circ_0004087 and SND1 will not affect each other reciprocally in PC-3 and DU145 cell lines (Additional file 2E and Additional file 2F).

The results mentioned above indicated that circ_0004087 can interact with SND1 protein via its RNA-binding domains SN3 and SN4.

### Circ_0004087 facilitates SND1-mediated transactivation of MYB

**Fig. 4:**
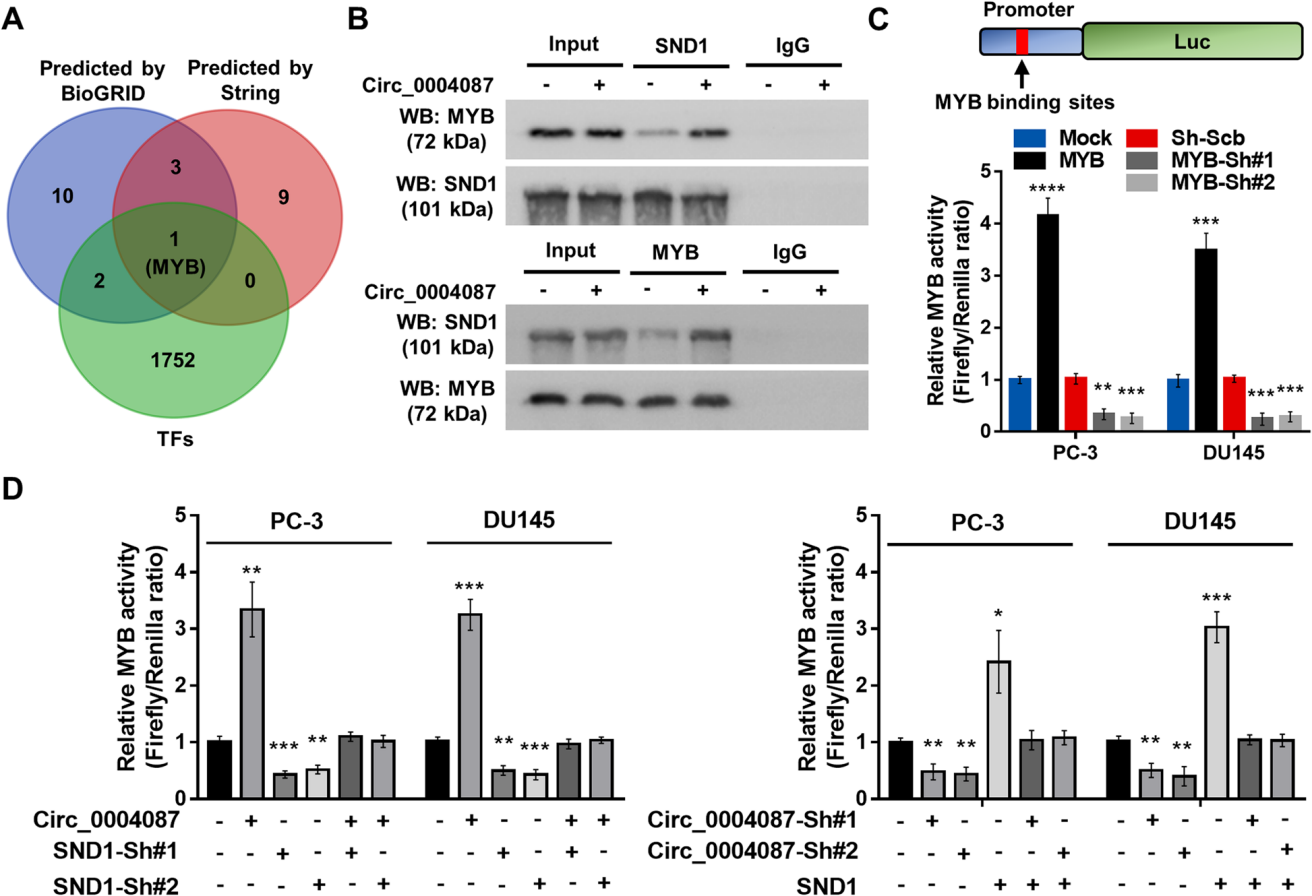
Circ_0004087 and SND1 co-regulated the MYB transcriptional activity in PCa cells. (**A)** Overlapping TFs with SND1-interacting proteins predicted by String (Score ≥ 0.9) and BioGRID (Evidence ≥ 2). **(B)** Co-IP and western blot assays revealing the interaction between SND1 and MYB. IgG was used as a negative control. **(C)** The construction of a dual-luciferase reporter vector containing canonical MYB binding sites (top). The transactivation of MYB in PC-3 and DU145 cells stably transfected with MYB-overexpressing or knocking-down vectors (bottom). **(D)** The relative MYB activity in PCa cells as indicated measured by dual-luciferase reporter assay. Statistical analysis is shown on the bar graphs. Data are presented as the mean ± SD of three independent experiments. * *P* < 0.05, ** *P* < 0.01, *** *P* < 0.001, **** *P* < 0.0001

SND1 has been identified as a transcriptional coactivator [30–32], compressive analysis using the protein interaction databases (STRING [33] and BioGRID [34]) and transcription factor database (GTRD [35], http://gtrd.biouml.org) revealed that MYB might be a potential SND1-interacting transcription factor (Fig. 4A). As predicted, Co-IP assay indicated that in PCa cells, MYB could be bonded by SND1, and the interaction was stronger following the overexpression of circ_0004087 (Fig. 4B). It has been reported that Pim-1 could bind with SND1 and stimulate MYB transcriptional activity in an SND1-dependent manner [36], so we explored the possibilities of circ_0004087 functionating in the same way. We performed the dual-luciferase reporter assay with a reporter containing the canonical MYB binding site which has been proven effective (Fig. 4C), the results showed that upregulated circ_0004087 could attenuate the decreased MYB transactivation induced by silencing of SND1 in PCa cells, vice versa (Fig. 4D).

### Circ_0004087 enhances the promoter activity of BUB1 in an SND1-dependent manner

**Fig. 5:**
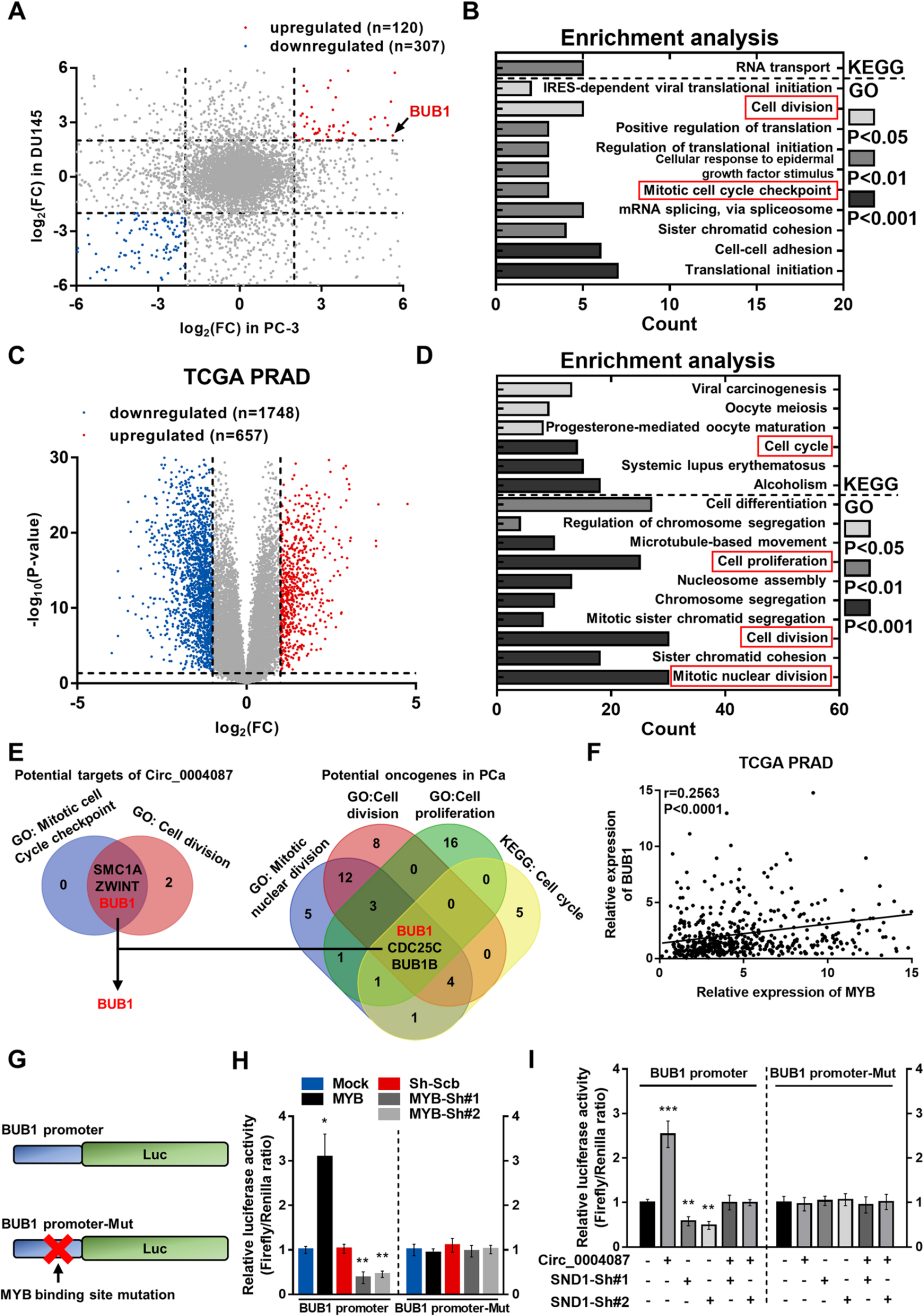
Circ_0004087 enhances BUB1 promoter activity in an SND1-dependent manner. (**A)** Differentially expressed genes in circ_0004087-overexpressing PCa cell lines. |log_2_(FC)|≥ 2, *P* < 0.05. **(B)** Enrichment analysis of potential targets of circ_0004087. **(C)** Differentially expressed genes in TCGA PRAD dataset. |log_2_(FC)|≥ 1, *P* < 0.05. **(D)** Enrichment analysis of potential oncogenes in PCa. **(E)** Overlapping cell cycle-related gene sets. **(F)** Correlation analysis for MYB and BUB1 expression in TCGA PRAD dataset. **(G)** Schematic diagram of dual-luciferase reporter vectors containing wild-type (BUB1 promoter) or mutant (BUB1 promoter-Mut) BUB1 promoter. **(H)** The activity of wild-type (BUB1 promoter) and mutant (BUB1 promoter-Mut) BUB1 promoter in PCa cells stably transfected with MYB-overexpressing or knocking-down vectors. **(I)** The activity of wild-type (BUB1 promoter) and mutant (BUB1 promoter-Mut) BUB1 promoter in PCa cells as indicated measured by dual-luciferase reporter assay. Statistical analysis is shown on the bar graphs. Data are presented as the mean ± SD of three independent experiments. * *P* < 0.05, ** *P* < 0.01, *** *P* < 0.001, **** *P* < 0.0001

To screen out the downstream targets of circ_0004087 in PCa, we conducted the RNA-seq in two stable circ_0004087-overexpressing PCa cell lines, including PC-3 and DU145. A total of 427 differentially expressed genes (DEGs) were detected in both two cell lines, with 120 upregulated genes and 307 downregulated genes [|log_2_(FC)|≥ 2, *P* < 0.05] (Fig. 5A). Notably, the upregulated genes were mainly enriched in cell cycle-related gene sets (Fig. 5B). We then explored the DEGs which might affect the development of prostate cancer using the TCGA PRAD dataset. Totally, 657 genes and 1748 genes were identified as being upregulated and downregulated [|log_2_(FC)|≥ 1, *P* < 0.05] respectively in tumor samples compared with normal samples (Fig. 5C). The enrichment analysis revealed that upregulated genes, in other words, the potential oncogenes, in PCa also mainly enriched in cell cycle-related gene sets (Fig. 5D). Overlapping the potential targets of circ_0004087 and the potential oncogenes in PCa, we identified BUB1 as a cell cycle-related oncogene in PCa which could be targeted by circ_0004087 (Fig. 5E). Furthermore, a transcription factor binding site (TFBS) of MYB was predicted in the region of BUB1 promoter via JASPAR database [37] (Additional file 6A), and the expression matrix acquired from TCGA PRAD revealed a positive correlation between them (Fig. 5F).

Considering the co-regulation of circ_0004087 and SND1 on MYB transcriptional activity, we then constructed dual-luciferase reporter vectors harboring the sequence of BUB1 promoter (BUB1 promoter) and the corresponding mutated sequence (BUB1 promoter-Mut) to uncover the possible relations among circ_0004087, SND1, and the transcriptional level of BUB1 (Fig. 5G). As expected, the promoter activity of BUB1, which could be enhanced by the ectopic expression of MYB, was promoted by the overexpression of circ_0004087 and suppressed by the knockdown of SND1 (Fig. 5H and I). Given the above, circ_0004087 can mediate the promoter activity of BUB1 in an SND1-dependent manner.

### BUB1 is upregulated in prostate cancer and renders prostate cancer cells the docetaxel resistance

**Fig. 6:**
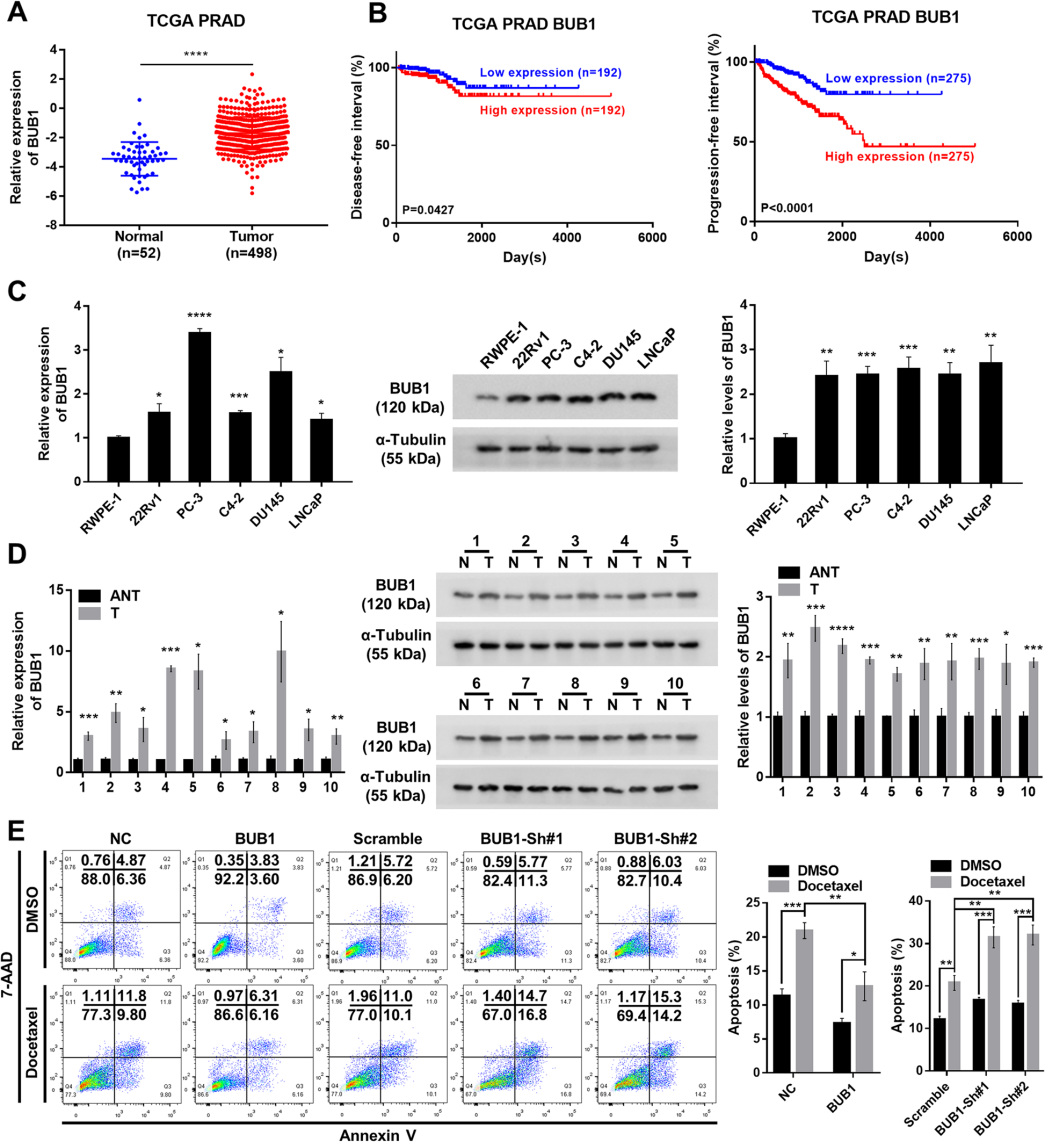
BUB1 enhanced the docetaxel resistance in PCa cells. (**A)** The expression pattern of BUB1 in TCGA PRAD database. **(B)** Kaplan–Meier analysis of disease-free interval (Left) and progression-free interval (Right) in PCa patients with high or low expression of BUB1. **(C)** Relative mRNA (Left) and protein (Right) levels of BUB1 in human prostatic epithelial cell line (RWPE-1) and PCa cell lines (22Rv1, PC-3, C4-2, DU145, and LNCaP). GAPDH and α-Tubulin were used as internal controls. **(D)** Relative mRNA (Left) and protein (Right) levels of BUB1 in 10 pairs of PCa and adjacent normal tissues. GAPDH and α-Tubulin were used as internal controls. ANT/N, Adjacent normal tissues; T, Tumor tissues. **(E)** The apoptosis rate of PCa cells expressing different BUB1 levels measured by flow cytometry. Cells were treated with 5 nM DTX for 48 h in this experiment. Cells went through the early apoptosis were marked with Annexin V-PE + /7-AAD-, cells went through the late apoptosis were marked with Annexin V-PE + /7-AAD + . NC, Negative Control. Statistical analysis is shown on the bar graphs. Data are presented as the mean ± SD of three independent experiments. * *P* < 0.05, ** *P* < 0.01, *** *P* < 0.001, **** *P* < 0.0001

To investigate the impact on clinical outcome of BUB1, which was significantly upregulated in PCa (Fig. 6A), we analyzed the disease-free interval and the progression-free interval of PCa patients acquired from TCGA PRAD dataset. Kaplan–Meier survival curves demonstrated that a high level of BUB1 was significantly associated with poor prognostic (Fig. 6B). Next, we performed qRT-PCR and western blot to further validate the higher mRNA and protein levels of BUB1 in PCa cell lines and clinical tumor tissues (Fig. 6C and D). Similar to circ_0004087 in function, the result of flow cytometry indicated that BUB1 boosted the DTX resistance in prostate cancer cells (Fig. 6E). Altogether, BUB1 is a potential tumor-promoting gene and partly renders prostate cancer cells the DTX resistance.

### Circ_0004087 and BUB1 co-contributes to the docetaxel resistance of prostate cancer cells

**Fig. 7:**
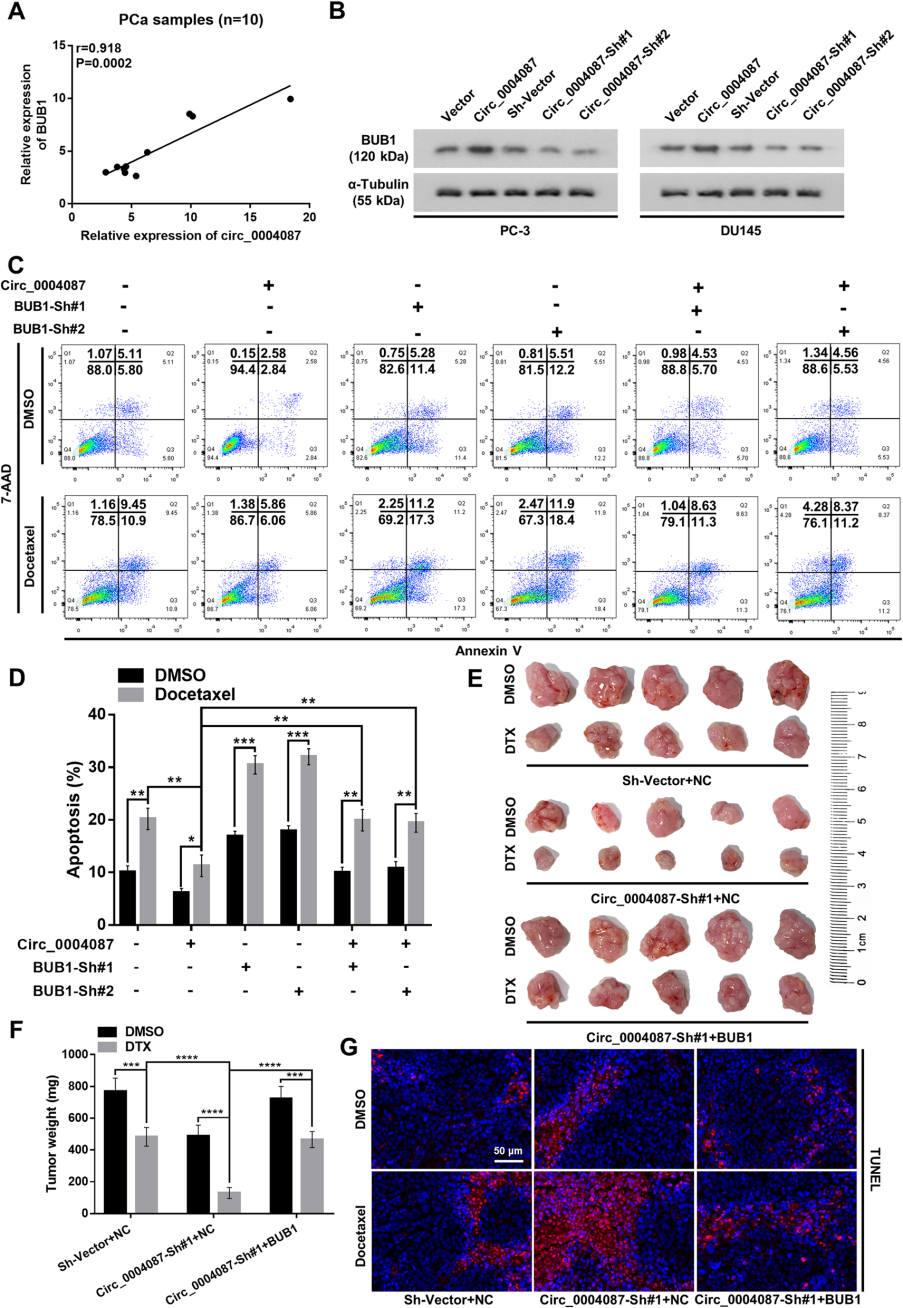
Circ_0004087 regulates docetaxel resistance of PCa cells by mediating BUB1 expression. (**A)** Correlation analysis for circ_0004087 and BUB1 mRNA levels in PCa samples. **(B)** Protein levels of BUB1 in circ_0004087-overexpressing or knocking-down PCa cell lines. α-Tubulin was used as an internal control. **(C-D)** The apoptosis rate of PCa cells as indicated. Cells were treated with 5 nM DTX for 48 h. Cells went through the early apoptosis were marked with Annexin V-PE + /7-AAD-, cells went through the late apoptosis were marked with Annexin V-PE + /7-AAD + . **(E)** Images of excised xenografts acquired using the digital single-lens reflex camera. **(F)** Tumor weights were measured on the day 30. **(G)** Immunohistochemical images of TUNEL staining on xenografts from each group 400 × . Statistical analysis is shown on the bar graphs. Data are presented as the mean ± SD of three independent experiments. * *P* < 0.05, ** *P* < 0.01, *** *P* < 0.001, **** *P* < 0.0001

For the reason that circ_0004087 could enhance the transcriptional level of BUB1 by prompting the combination of SND1 and MYB, we performed qRT-PCR and western blot to determine the modulation of circ_0004087 upon BUB1 mRNA and protein. The data showed that both mRNA and protein levels of BUB1 increased following the upregulation of circ_0004087 in PCa, vice versa (Fig. 7A and B, Additional file 6B). Moreover, the DTX resistance of PCa cells contributed by increased circ_0004087 could be neutralized by decreased BUB1 (Fig. 7C and D). In accordance with the tendency observed in vitro, in vivo tumor growth assay showed that the hypersensitivity to DTX in circ_0004087-knocking-down group could be weakened by upregulating BUB1 (Fig. 7E and F). Also, the results of TUNEL staining assay indicated that circ_0004087 and BUB1 co-regulated the DTX-inducing apoptosis of PCa cells (Fig. 7G).

### Knockdown of circ_0004087 disrupts the docetaxel resistance of PCa cells by breaking down the correction mechanism of mitosis

**Fig. 8:**
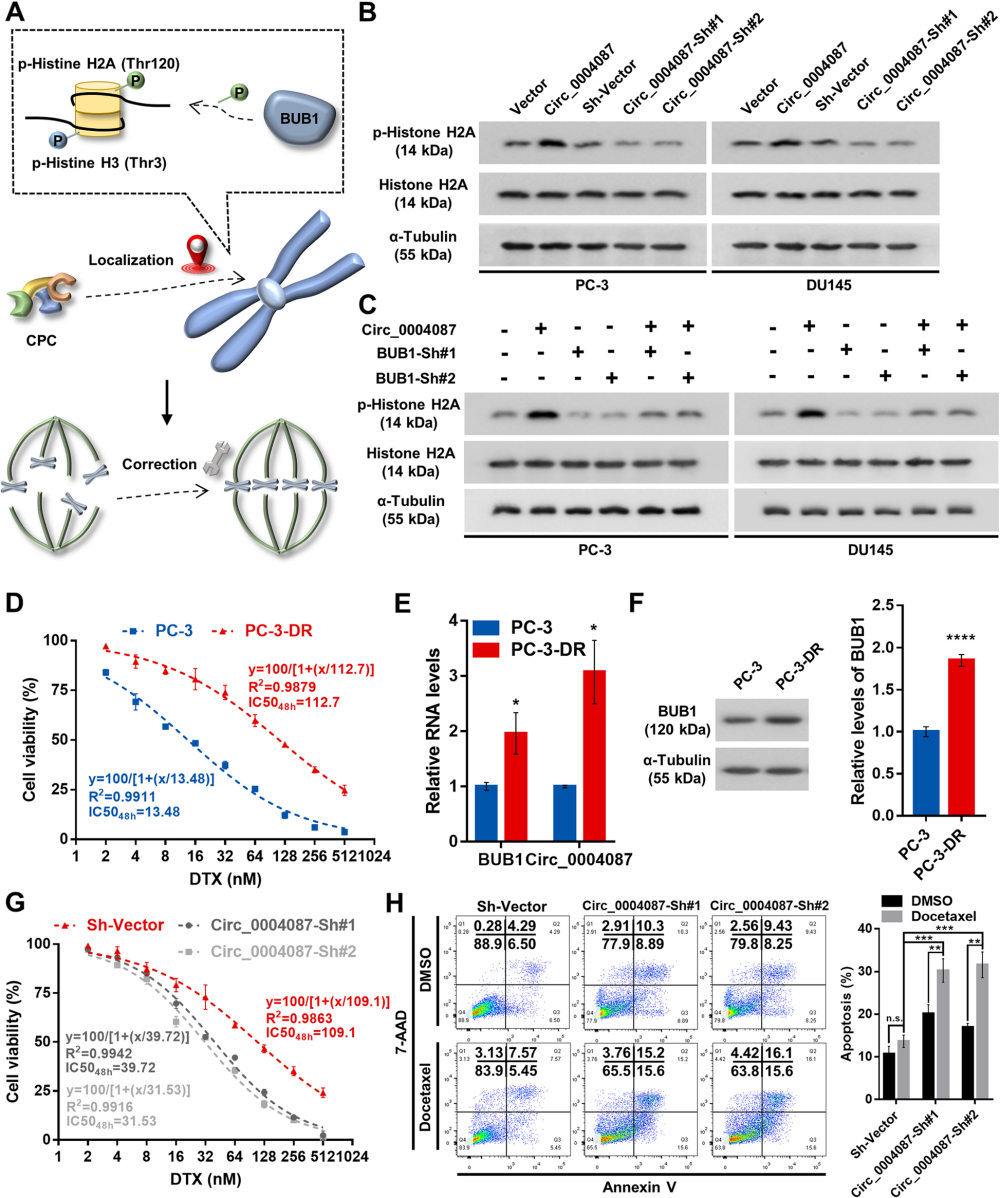
Knocking down circ_0004087 impairs the docetaxel resistance of PCa cells. (**A)** BUB1 guarantees the error-free mitosis by correctly localizing CPC to centromeres. **(B)** The phosphorylation level of Histone H2A in circ_0004087-overexpressing or knocking-down PCa cell lines. α-Tubulin was used as an internal control. **(C)** The phosphorylation level of Histone H2A in PCa cells as indicated. α-Tubulin was used as an internal control. **(D)** Determination of IC50_48h_ values for parental (PC-3) and DTX-resistant (PC-3-DR) PC-3 cells. **(E)** Levels of BUB1 mRNA and circ_0004087 in parental (PC-3) and DTX-resistant (PC-3-DR) PC-3 cells. **(F)** BUB1 protein expression in parental (PC-3) and DTX-resistant (PC-3-DR) PC-3 cells. **(G)** Determination of IC50_48h_ values for DTX treatment in PC-3-DR cells which were stably transfected with sh-vector, circ_0004087-sh#1, and circ_0004087-sh#2. **(H)** The apoptosis rate of PC-3-DR cells with downregulated circ_0004087. Cells were treated with 10 nM DTX for 48 h in this experiment. Cells went through the early apoptosis were marked with Annexin V-PE + /7-AAD-, cells went through the late apoptosis were marked with Annexin V-PE + /7-AAD + . Statistical analysis is shown on the bar graphs. Data are presented as the mean ± SD of three independent experiments. * *P* < 0.05, ** *P* < 0.01, *** *P* < 0.001, **** *P* < 0.0001

BUB1 has been reported to phosphorylate histone H2A at T120, and recruit the chromosomal passenger complex (CPC) to centromeres [38–41], which plays a crucial role in error-free mitosis [18] (Fig. 8A). On the other hand, knocking down the components of CPC could weaken the chemoresistance in several cancers [23, 42]. So, we next investigated the co-effect of circ_0004087 and BUB1 on the phosphorylation of Histone H2A, which has been well proved to contribution to the recruitment of CPC to centromeres. The western blot assay revealed a higher level of phosphorylated histone H2A in circ_0004087-overexpressing PCa cells (Fig. 8B and Additional file 6C), which could be attenuated by knocking down the expression of BUB1 (Fig. 8C and Additional file 6D).

To further determine the role of circ_0004087 in regulating the DTX resistance of PCa cells, we constructed DTX-resistant PC-3 cells (PC-3-DR) (Fig. 8D), and found that both circ_0004087 and BUB1 were significantly upregulated in PC-3-DR (Fig. 8E and F). Furthermore, by measuring the half maximal inhibitory concentration (IC50) and apoptosis rate, we discovered that silencing of circ_0004087 could partly impair the property of DTX resistance in PCa cells (Fig. 8G and H).

**Fig. 9:**
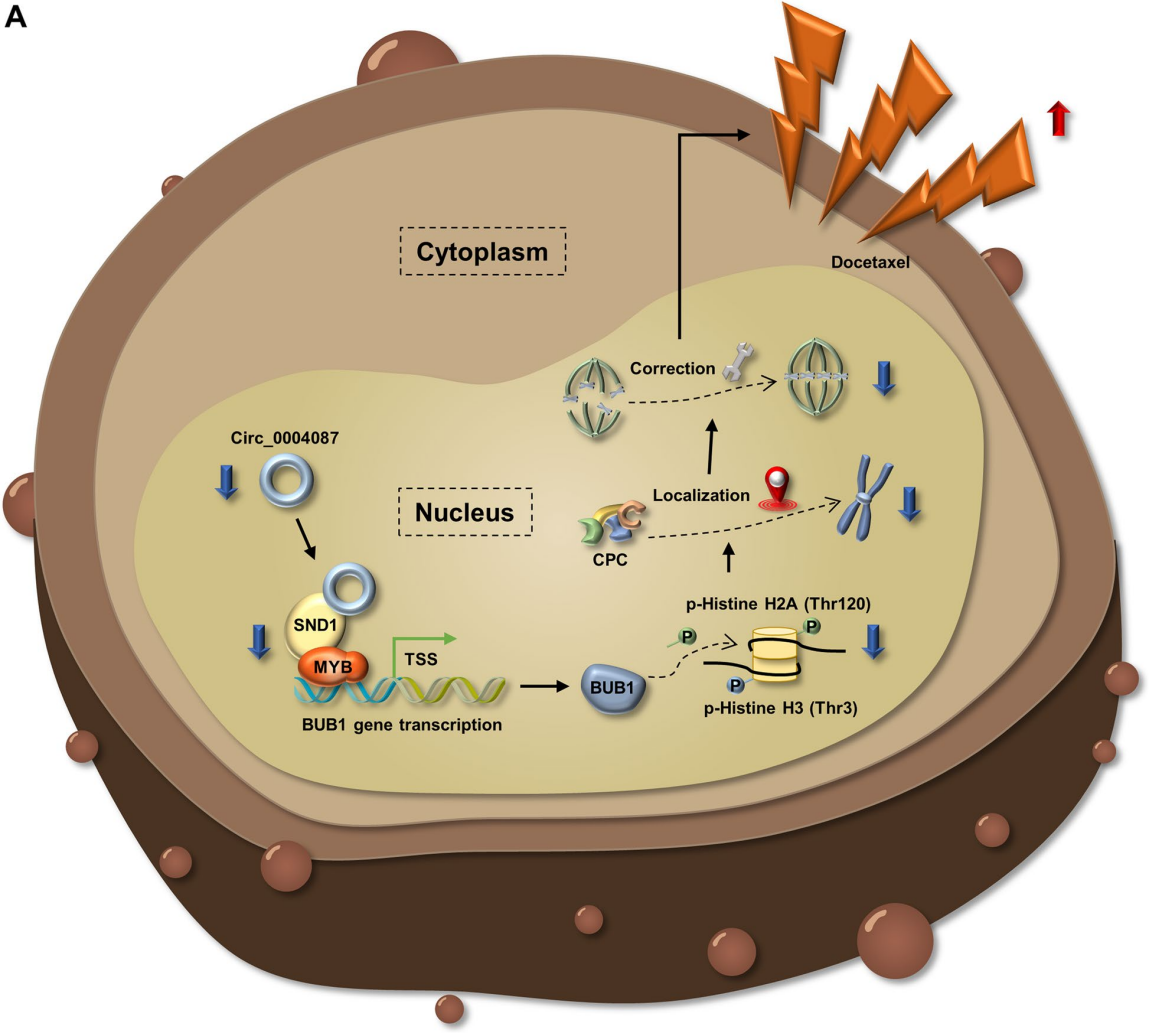
The schematic diagram of circ_0004087 in regulating docetaxel resistance. (**A)** In an SND1-dependent manner, downregulating circ_0004087 suppresses the transactivation of MYB and reduces BUB1 expression, further augments the effect of docetaxel on PCa cells by breaking down the correction mechanism of mitosis

In summary, our results revealed that in an SND1-dependent manner, knockdown of circ_0004087 suppresses the transactivation of MYB and reduces BUB1 expression, further disrupts the DTX resistance of PCa cells by breaking down the error correction mechanism of mitosis, suggesting that circ_0004087 is a potential therapeutic target to improve DTX chemosensitivity in prostate cancer (Fig. 9A).

## Discussion

Prostate cancer has been one of the leading causes of cancer-related deaths in men worldwide [1]. Moreover, the incidence of prostate cancer in China is gradually increasing these years [43, 44]. Despite the initial therapeutic effect of androgen deprivation therapy on PCa patient survival, most cases eventually progress to CRPC, making it a global challenge [45–47]. DTX, a chemotherapeutic agent blocking tubulin depolymerization and inhibiting microtubule dynamics [48, 49], is used as the standard first-line therapy for mCRPC patients for decade [5]. However, patients usually develop DTX resistance finally [50]. CircRNAs are reported to be stable, conserved, and tissue-specific in mammalian cells [8, 9]. Recently, growing evidence uncovered the close association between circRNAs and DTX resistance. Chen et al. found that circ_0006404 and circ_0000735 could co-regulate the response of ovarian cancer to DTX treatment [51]. Another study revealed that blocking circ_0014130 could weaken the drug resistance of DTX-resistant non-small cell lung cancer via regulating miR-545-3p-YAP1 axis [52]. CircRNA CRIM1 was reported to promote nasopharyngeal carcinoma DTX chemoresistance through upregulating FOXQ1 [53].

In our study, we uncovered the promoting effect of circ_0004087 on DTX resistance in PCa and identified a higher level of circ_0004087 in DTX-resistant PCa cells. Mechanistically, circ_0004087 enhanced transactivation of MYB in an SND1-dependent manner and increased protein expression of BUB1, further promoting the repair function of CPC in mitosis, thus contributing to the DTX resistance of PCa.

Previous studies have shown that some circRNAs could modulate the DTX sensitivity of PCa by sponging miRNAs. The study performed by Gao et al. revealed that knocking down circ_0000735 sensitized PCa to DTX treatment via sponging miR-7 [16]. Besides, exosomal circ-XIAP was identified to promote DTX resistance of PCa by regulating miR-1182/TPD52 axis [15]. Although miRNA sponge is the predominant mechanism by which circRNA functions, it is well recognized that circRNAs perform functions in some other ways, including protein synthesis, protein scaffold, protein sponge, and transcription factor recruitment [26]. Herein, RNA pull-down assay and mass spectrometry analysis confirmed that circ_0004087 modulated DTX sensitivity by forming a specific circRNA-protein complex (circRNPs) with SND1 protein. Also, we constructed Flag-tagged protein truncations and confirmed binding domains of SND1 to circ_0004087. Our results indicated that circ_0004087 combined with SN3 and SN4 domains of SND1. This finding was supported by research performed by Li et al., who suggested that nucleic acid substrates were bound to SND1 via SN3 and SN4 domains, and a minimum of two tandem SN domains were required for efficient RNA binding [29].

SND1, a transcriptional coactivator, has been reported to interact with various transcription factors, such as STAT5, STAT6, MYB, and E2F1 [30–32, 36]. Previous study showed that the transcriptional coactivation activity of SND1 could be modulated by upstream regulator. Leverson et al. discovered that protein kinase Pim-1 could interact with SND1 and form a stable complex in animal cells. This combination subsequently altered the affinity of SND1 for transcription factor MYB, and finally enhanced MYB transcriptional activity [36]. However, the effect of circRNAs on transcriptional coactivation function of SND1 in cancer progression remains to be investigated. In the present study, we revealed that circ_0004087 bonded SN3 and SN4 domains of SND1 protein, further increasing its transcriptional coactivation activity and augmenting the transactivation of MYB. We believe that the combination with circ_0004087 might lead to the alteration of SND1 protein structure, which could facilitate its interaction with MYB. However, the underlying mechanisms warranting further investigation.

DTX, a microtubule-targeting agent, has been broadly applied to treating multiple solid tumors, however, their clinical efficacy is often compromised by the emergence of drug resistance [54, 55]. Considering that the mode of action of DTX is to increase the rate of attachment errors, and that inactivation of cell-cycle checkpoints can cause fatal injury on tumor cells, a reliable hypothesis was put forward: inhibition of the spindle attachment error correction mechanisms like CPC, could improve the efficacy of DTX and reverse the drug resistance [22]. As expected, this hypothesis was reinforced by some existed researches. Survivin, together with INCENP, borealin, and AURKB to form CPC, has been reported to be overexpressed in many cancers and enable chemotherapy resistance. The reduction of survivin by siRNA could reverse drug resistance [23]. In addition, another component of CPC, AURKB, has been found to contribute to drug resistance in several tumors. The therapeutic strategy targeting AURKB successfully overcomes the chemoresistance in different malignancies [42]. Here in, we tried to disclose the connection between circRNAs and CPC, which may facilitate the treatment of chemo-resistant PCa. Our study suggested that, in PCa, the level of circ_0004087 was positively correlated with the expression of BUB1, which has been well proved to phosphorylate Histone H2A, contribute to the recruitment of the Shugoshins to centromeres, and eventually lead to the localization of CPC on centromeres [38–41]. In this way, downregulating circ_0004087 would prevent the recruitment of CPC to centromeres by suppressing BUB1, and subsequently impair the error correction mechanism in mitosis partly. Furthermore, by measuring apoptosis rate, we discovered that silencing of circ_0004087 could exert a synergistic effect with DTX and partly impair the property of DTX resistance in the PC-3-DR cell line. These findings were supported by a study performed by Siemeister et al., they indicated that BUB1 kinase inactivation could mitigate the resolution capability upon spindle attachment errors and bring about an increased rate of chromosome alignment defects, in particular, in presence of attachment error–inducing agents such as microtubule stabilizer paclitaxel [22].

## Conclusions

In conclusion, our study uncovered the potential interaction between circRNAs and the mitosis error correction mechanism, providing a brand-new approach in treating with the DTX-resistant PCa. These findings suggested the crucial role of circ_0004087/SND1/MYB/BUB1 axis in the development of DTX-resistant prostate cancer. Hopefully, our exploration of the underlying mechanism of circ_0004087 will provide prospective targets for DTX-resistant prostate cancer.

## Supplementary Information

**Additional file 1.** The sequences of primers, oligonucleotides and probes used in this study.

**Additional file 2.** (A) Schematic diagram showing the probe specifically designed for circ_0004087. (B) The efficiency of circ_0004087 probe verified by qRT-PCR. (C) The specificity of circ_0004087 probe determined by agarose gel electrophoresis. (D) Subcellular distribution of SND1, ACTN4, BZW1, CALR, ECH1, and PDIA6 in PCa cells. Nuclei were stained with DAPI. 1600×. (E) Relative expression of circ_0004087 in indicated cell lines. (F) Protein levels of SND1 in indicated cells.

**Additional file 3.** Mass spectrometry assay of proteins pulled down by circ_0004087.

**Additional file 4.** RNA-binding proteins.

**Additional file 5.** Interaction propensity of SND1 protein residue to circ_0004087 predicted by catRAPID algorithm.

**Additional file 6.** (A) TFBS of MYB in BUB1 promoter predicted by JASPAR (Relative profile score threshold = 90%). (B) Protein levels of BUB1 in circ_0004087-overexpressing or knocking-down PCa cell lines. a-Tubulin was used as an internal control. (C) The phosphorylation level of Histone H2A in circ_0004087-overexpressing or knocking-down PCa cell lines. a-Tubulin was used as an internal control. (D) The phosphorylation level of Histone H2A in PCa cells as indicated. a-Tubulin was used as an internal control. 

## Data Availability

The datasets used and/or analysed during the current study are available from the corresponding author on reasonable request.

## Abbreviations

CircRNAs: Circular RNAs
PCa: Prostate cancer
SND1: Staphylococcal Nuclease And Tudor Domain Containing 1
BUB1: BUB1 Mitotic Checkpoint Serine/Threonine Kinase
RIP: RNA immunoprecipitation
TCGA: The Cancer Genome Atlas
DTX: Docetaxel
FISH: Fluorescent in situ hybridization
CCK-8: Cell Counting Kit-8
IHC: Immunohistochemistry
ShRNAs: Small hairpin RNAs
CPC: Chromosomal passenger complex
CRPC: Castration-resistant prostate cancer
