# Supplementary figures and images for: Circ_0004087 interaction with SND1 promotes docetaxel resistance in prostate cancer by boosting the mitosis error correction mechanism
Source: J Exp Clin Cancer Res. 2022 Jun 3;41:194. doi: 10.1186/s13046-022-02404-3 (PMC9166435; doi:10.1186/s13046-022-02404-3)

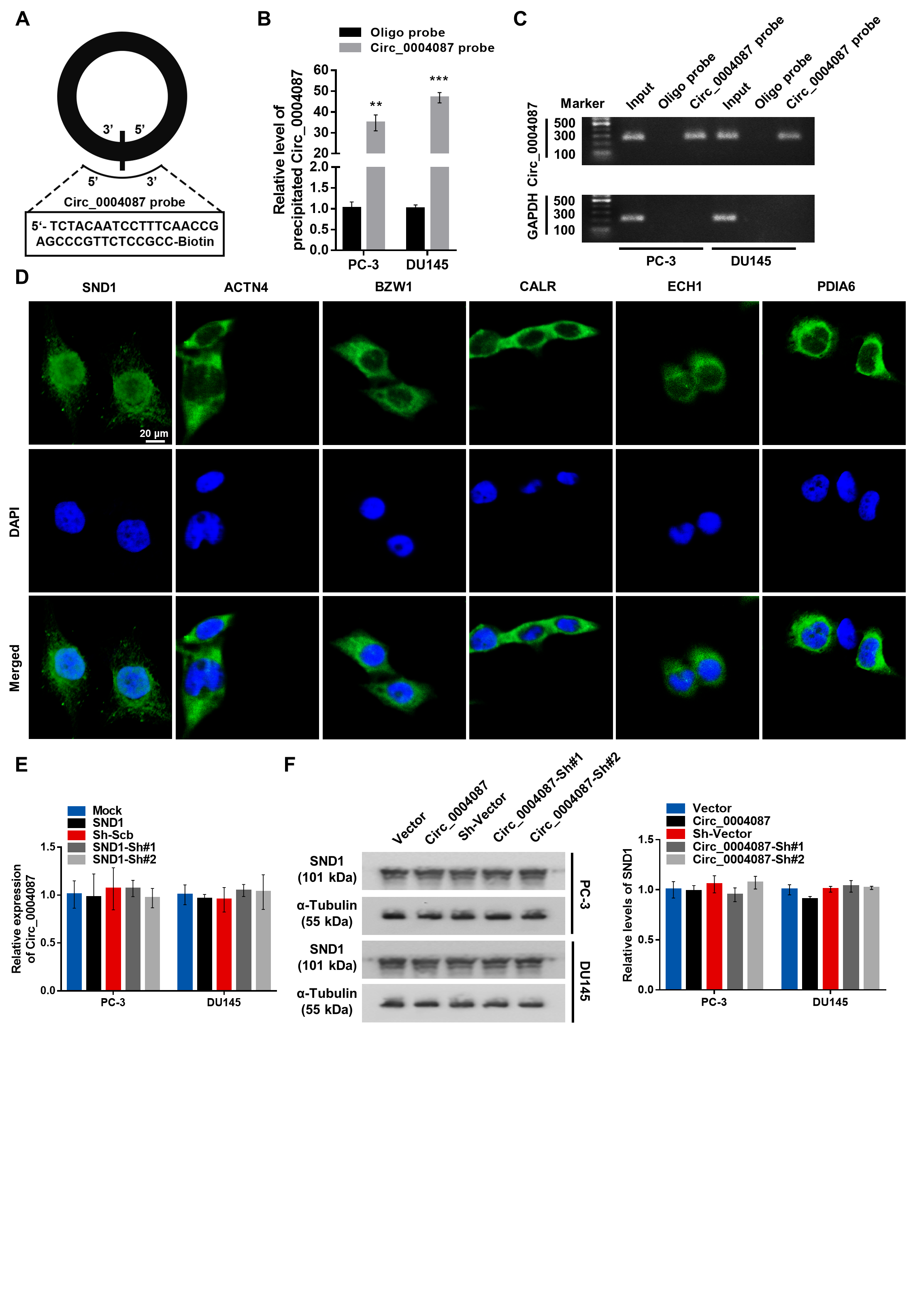

Supplement: Supplementary file 2 — Additional file 2. (A) Schematic diagram showing the probe specifically designed for circ_0004087. (B) The efficiency of circ_0004087 probe verified by qRT-PCR. (C) The specificity of circ_0004087 probe determined by agarose gel electrophoresis. (D) Subcellular distribution of SND1, ACTN4, BZW1, CALR, ECH1, and PDIA6 in PCa cells. Nuclei were stained with DAPI. 1600×. (E) Relative expression of circ_0004087 in indicated cell lines. (F) Protein levels of SND1 in indicated cells. [file 13046_2022_2404_MOESM2_ESM.tif]

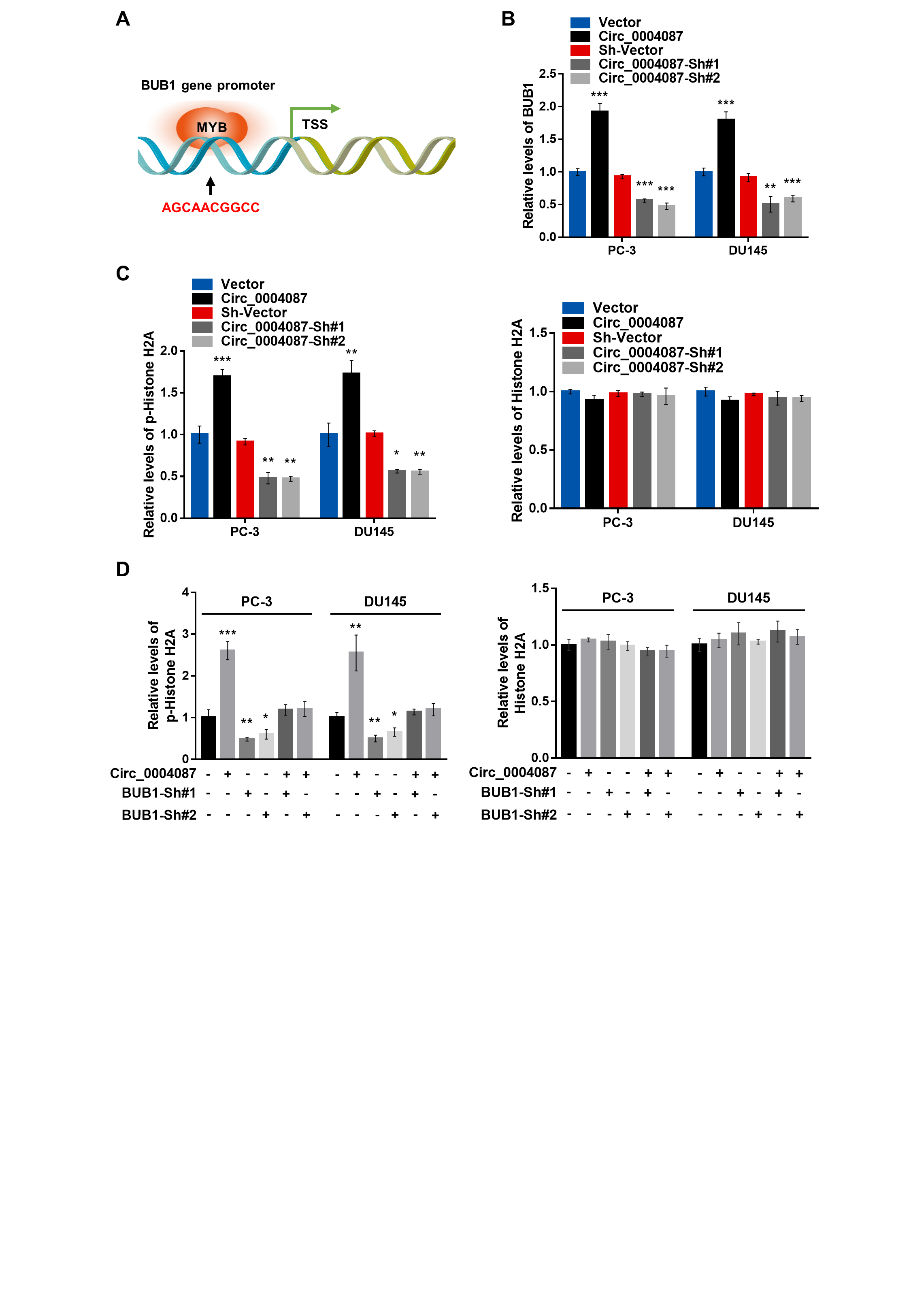

Supplement: Supplementary file 6 — Additional file 6. (A) TFBS of MYB in BUB1 promoter predicted by JASPAR (Relative profile score threshold = 90%). (B) Protein levels of BUB1 in circ_0004087-overexpressing or knocking-down PCa cell lines. a-Tubulin was used as an internal control. (C) The phosphorylation level of Histone H2A in circ_0004087-overexpressing or knocking-down PCa cell lines. a-Tubulin was used as an internal control. (D) The phosphorylation level of Histone H2A in PCa cells as indicated. a-Tubulin was used as an internal control. [file 13046_2022_2404_MOESM6_ESM.tif]
